# From Inactivity to Activity: Passive Wheelchair Bike Rides Increase Trapezius Muscle Activity in Non-Ambulant Youth with Disabilities

**DOI:** 10.3390/children12060792

**Published:** 2025-06-17

**Authors:** Lisa Musso-Daury, Celia García-Chico, Susana López-Ortiz, Saúl Peñín-Grandes, Diego del Pozo-González, Rosa Ana Sánchez-García, Laura Marín-Varela, Carmen Matey-Rodríguez, Alejandro Santos-Lozano

**Affiliations:** 1 i+HeALTH Strategic Research Group, Department of Health Sciences, Miguel de Cervantes European University, 47012 Valladolid, Spain; lmusso@uemc.es (L.M.-D.); cgarciac@uemc.es (C.G.-C.); spenin@uemc.es (S.P.-G.); cmatey@uemc.es (C.M.-R.); asantos@uemc.es (A.S.-L.); 2 Colegio de Educación Especial Pino de Obregón, Fundación Personas, 47008 Valladolid, Spain; 3 Research Institute of the Hospital 12 de Octubre (‘imas12’), 28041 Madrid, Spain

**Keywords:** child, adolescent, cerebral palsy, bicycling, exercise

## Abstract

**Background/Objectives**: Children at Gross Motor Function Classification System (GMFCS) levels IV and V experience severe motor impairments, yet the effects of passive wheelchair rides on their physiological parameters remain unexplored. This study aimed to examine the acute physiological response to passive bike in non-ambulant children with physical disabilities. **Methods**: This quasi-experimental study included 24 non-ambulant participants with cognitive impairments (6–21 years old, 50% female). After a 10-min rest, participants underwent a 10-min passive wheelchair bike. Muscle activity, oxygen consumption, and heart rate variability were assessed. **Results**: Passive bike rides significantly increased muscle activity in the right upper (*p* = 0.050), left upper (*p* = 0.008), and left lower trapezius (*p* = 0.038), with increases of 97–112%. However, no significant changes were observed in oxygen consumption or cardiorespiratory parameters. **Conclusions**: This study suggests that passive wheelchair bike rides increase trapezius muscle activity in children with severe disabilities at GMFCS levels IV and V, offering potential benefits for this population.

## 1. Introduction

Children and adolescents who are classified in levels IV and V according to the Gross Motor Function Classification System (GMFCS) [1] experience significant physical impairments that difficult autonomous or assisted walking. As they age, these physical disabilities progress and further limit their activity, participation and general well-being [2]. They have a significant impact on their health and quality of life, so targeted interventions are required to address their specific needs. Physical activity is essential for the health and well-being of all children and adolescents, including those with disabilities [3]. The World Health Organization (WHO) highlights that children with disabilities should be as physically active as their non-disabled peers, recommending at least 60 min of moderate-to-vigorous physical activity per day [4]. However, physical disability is often a significant barrier to meeting these recommendations, potentially exacerbating health inequalities [5]. The International Classification of Functioning, Disability, and Health for Children and Youth (ICF-CY), deriving of the International Classification of Functioning, Disability, and Health (ICF) developed by the WHO [6] provides a comprehensive framework for understanding and addressing the complex interplay between a child’s health condition and their environment. More recently, the “F-words” (Function, Family, Fitness, Fun, Friends, and Future) for child development approach, based on ICF framework, integrate important themes to children and young people’s functioning and well-being, with “Fitness” specifically emphasizing the need for adapted physical activities [7,8]. For children and adolescents who are non-ambulant (i.e., GMFCS levels IV and V), adapted physical activities are essential. The aim of this emphasis on fitness is to enhance their body structure and function while also improving their overall health and participation in daily activities [9]. Among the adapted activities that can support the “F-words approach”, passive ride bike stands out as a promising intervention.

Therefore, it is crucial to develop and implement strategies that facilitate the participation of children and adolescents with severe motor impairments in physical activities. For that reason, enhancing cardiorespiratory fitness, muscle strength, and cardiovascular health is essential for children with severe physical impairments [10,11]. Consequently, muscle activity [12], oxygen consumption (VO_2_) [13], and heart rate variability (HRV) [14] are key physiological variables that can be measured to assess these aspects. Even if the activity is passive, assessing its acute physiological effects in this population is essential, as it provides objective insights into the minimal thresholds required to elicit neuromuscular or cardiorespiratory responses in individuals with severely limited motor capacity.

Maximum VO_2_, which measures the maximum amount of oxygen an individual can utilize during exercise, is a critical indicator of cardiovascular fitness and overall aerobic capacity [13]. Although passive activities are generally assumed to have minimal impact on cardiorespiratory function, there is limited evidence assessing VO_2_ responses in individuals with severe cerebral palsy [15]. Regarding muscle activity, often assessed through surface electromyography (sEMG), it provides insights into neuromuscular function and muscle activation patterns [16]. Enhancing muscle strength is crucial for preventing muscle atrophy and contractures [17], which are common in children with severe physical impairments [18]. Increased muscle strength can lead to better stability, functional independence, and an overall improvement in quality of life. In the other hand, HRV represents the variation in time intervals between heartbeats and is a marker of autonomic nervous system function and cardiovascular health [14]. Higher HRV is associated with better autonomic regulation and lower stress levels, while lower HRV is often linked to increased cardiovascular risk and reduced parasympathetic activity [19]. Improving HRV can enhance autonomic function, reduce stress, and improve overall cardiovascular health [20]. However, given their limited mobility, traditional forms of physical exercise are often not feasible, requiring innovative approaches adapted to their abilities [21].

Although evidence in non-ambulant children and adolescents is limited, passive wheelchair bike ride could improve key physiological and motor functions. A 6-week program of static cycling (with support for the trunk, wrists, and feet) has already shown significant improvements in cycling ability [22] and gross motor function in the short term [23]. Adaptive activities like passive ride offer new opportunities to enhance motor skills and promote physical activity [24]. However, applying this bike activity to all children and adolescents can be challenging. Passive wheelchair bike, which uses a modified tandem bike with a detachable wheelchair at the front, allows individuals with limited mobility to enjoy a ride while a caregiver pedals and steers from behind [25]. To date, no studies have evaluated the acute effects of passive ride on muscle activity, VO_2_, and HRV in children at GMFCS levels IV and V. Therefore, this study aims to investigate the acute physiological response to passive bike ride in this population.

## 2. Materials and Methods

### 2.1. Study Design

This quasi-experimental study was conducted between February and March 2024, after obtaining ethical approval from Human Ethics Committee of the Miguel de Cervantes European University (Ethics approval number: 6586). The trial was registered at OSF platform: https://doi.org/10.17605/OSF.IO/97VMA (accessed on 5 June 2025). The legal tutor signed the consent form to allow the children or adolescents to participate in the study. Passive wheelchair bikes were provided by the institution where the study was conducted.

### 2.2. Study Participants

Participants were identified and recruited at the Special Education School “El Pino de Obregón”, Valladolid, Spain. Invitations to participate were advertised by contacting the physiotherapists at the center. Participation criteria included children and adolescents with physical disability aged from 6 to 21 years old (inclusive) who were non-ambulant (i.e., GMFCS levels IV or V). Although developed for individuals with cerebral palsy, the GMFCS was used in this study to classify gross motor function of all children and adolescents. Due to the hight level of comorbidities on this population [26], cognitive impairment, epilepsia and physical deformities were not considered as exclusion criteria. Medical clearance and written consent were obtained from their legal tutors. Exclusion criteria included medical conditions that might have affected the ability to participate safely and musculoskeletal surgery (or other major surgery) in the six months prior to the intervention.

### 2.3. Sample Size

Based on previous research [27] that reported a large effect size (1.58 ± 0.46), representing a percentage increase in muscle activity for the sEMG variable from a resting state among children and adolescents to sitting and standing with support, a sample size of at least 17 participants was required to detect a significant effect (95% power, 2-tailed *p* < 0.05). In the present study, a total of 24 participants were included in the final analysis.

### 2.4. Passive Wheelchair Bike Ride Intervention

The intervention consisted of a 10-min seated rest (baseline values) followed by a 10-min passive wheelchair bike ride (i.e., 500 m per lap at medium speed of 10 ± 2 km/h), during which physiological values were recorded (during-activity values). The intervention was delivered by three physiotherapists from the center, and outcomes were assessed by external researchers.

The intervention used two different types of passive wheelchair bikes. The first type had a platform designed to accommodate participants’ wheelchairs so that they could participate without needing to transfer from their chairs (VeloPlus wheelchair bike, Van Raam). The second type had an integrated chair with headrest and customisable attachments (OPair wheelchair bike, Van Raam). Both bikes were electric, ensuring the participants’ safety during the activity. The school physiotherapist determined the use of each type of bike depending on participants capability and safety issues. Participants were allocated in the platform bike with their own chair if they couldn’t handle the integrated chair bike due to special needs for posture position.

### 2.5. Outcome Measures

The primary outcome was the trapezius muscle activity while secondary outcomes included cardiorespiratory response and autonomic nervous system response. All outcomes were measured at rest and during the passive wheelchair bike ride.

#### 2.5.1. Muscle Activity Measurement

Muscle activity of the left upper trapezius (LUT), right upper trapezius (RUT), left lower trapezius (LLT) and right lower trapezius (RLT) muscles were measured using sEMG. Before electrode placement, the area was cleaned with isopropyl alcohol [28]. One set of surface electrodes (Ag/AgCl, Skintact, Austria), composed of two measuring electrodes and a differential electrode, was placed longitudinally to the muscle fibers direction. An inter-electrode distance of 2 cm was maintained, and the reference electrode was placed on the acromion. The Surface ElectroMyoGraphy for the Non-Invasive Assessment of Muscles (SENIAM) [29] recommendations were followed for electrode placement.

Muscle activity assessment was measured by the wearable sEMG system mDurance© (mDurance Solutions SL, Granada, Spain). Two sEMG units (Realtime Technologies Ltd., Dublin, Ireland) were used, which are bipolar sEMG sensors for the acquisition of superficial muscle activity. Each sensor consists of two sEMG channels, with a sampling rate of 1024 Hz. Shimmer applies a bandwidth of 8.4 kHz, with an sEMG signal resolution of 24 bits and overall amplification of 100–10,000 V/V. Through Bluetooth 4.0 signals, both Shimmer units connected to the mDurance© app on a mobile phone. The mDurance Android application received data from the Shimmer units and sent it to a cloud service.

#### 2.5.2. Cardiorespiratory Response Measurement

The cardiorespiratory responses were measured with the VO_2_ Master metabolic analyzer (VO_2_ Master, Vernon, Canada), which was connected to the VO_2_ Master© mobile application via Bluetooth 4.0. The analyzer was attached to a Hans Rudolph 7450 V2 over-the-nose mask using the Rest Attachment, which connects the analyzer to the mask and includes an exhaust port for airflow. The mask was secured to the participant’s face with soft headgear. A disposable filter was placed between the user piece and the analyzer according to the manufacturer’s instructions and replaced after each test to ensure hygiene and measurement accuracy.

The VO_2_-Master device works with a passive gas sampling system equipped with an oxygen (O_2_) sensor and a flow sensor. On start-up, the device automatically calibrates itself to the ambient conditions (i.e., gas concentrations, temperature, humidity and air pressure), although it does not calibrate against reference gas mixtures. After calibration according to the manufacturer’s guidelines, the device was attached to each participant. The system measured ventilation (VE), respiratory rate, tidal volume, VO_2_ and oxygen equivalent, all of which were recorded on a breath-by-breath basis.

#### 2.5.3. Autonomic Nervous System Response Measurement

Autonomic response was measured before and during the intervention using the commercially available device Polar H10 heart rate (HR) sensor chest strap (Polar Electro Oy, Kempele, Finland) connected with the validated Elite HRV© app (Elite HRV Inc., Asheville, NC, USA). Each participant was fitted with this device, which collected and processed HRV measurements by detecting the electrical signals of the heart. Kubios HRV Scientific Little software (version 4.1.1, Kubios, Ltd., Kuopio, Finland) was used to analyze the collected data. After removing the detected artifacts with medium threshold of beats corrections, the HR and the time domain measures of HRV that were analyzed were standard deviation of normal-to-normal intervals (SDNN), Root Mean Square of the Successive Differences (RMSSD), and standard deviation of the heart rate (STD HR). Moreover, the frequency measurements of very low frequency (VLF, 0.0033–0.04 Hz), low frequency (LF, 0.04–0.15 Hz) and high frequency (HF, 0.15–0.4 Hz) and was obtained.

### 2.6. Data Reduction and Statistical Analysis

The mDurance© cloud service stored, filtered, and analyzed the sEMG signals, generating reports after manually excluding the first and last minute of each recording. Therefore, we analyzed the 8 central minutes of the resting period and the 8 central minutes of the bike ride period. The same process was applied to the VO2 Master device© and Elite HRV©, ensuring synchronization of the same 8-min measurement for each participant.

Data were presented as mean ± standard deviation (SD) and median (Quartile 1–Quartile 3). Shapiro-Wilk Normality test was used to assess the normality of all variables. Paired samples test analysis of variance with parametric variables and Wilcoxon when non-parametric variables were conducted to examine the effects of the intervention on the outcomes between the conditions (i.e., resting time and passive wheelchair bike ride).

Additionally, a sensitivity sub-analysis was performed to compare potential differences between children classified as GMFCS level IV and level V, evaluating whether outcomes varied based on group classification. For parametric data, a repeated measures mixed ANOVA was conducted, with time as the within-subjects factor and GMFCS group as the between-subjects factor. For non-parametric data, within-group comparisons were evaluated using the Wilcoxon signed-rank test for paired observations. Between-group differences were examined with the Mann-Whitney U test. Interaction effects were tested using a Mixed Model incorporating rank-transformed data. To reduce the risk of type I error, posthoc analyses by group were only performed with the Bonferroni test when a significant interaction (group-by-time) effect was observed.

All analyses were performed using the Statistical Package for Social Sciences (SPSS) program, version 20.0 for Windows. Significance levels were set at a *p*-value *≤ 0.05* for all variables, except for those related to HRV. For HRV-related variables, a Bonferroni correction was applied to account for multiple comparisons, adjusting the threshold for significance according to the number of variables analyzed (i.e., 16), resulting in a corrected significance level of *0.003* (*p*-value ≤ 0.05/16).

## 3. Results

### 3.1. Participants

**Figure 1:**
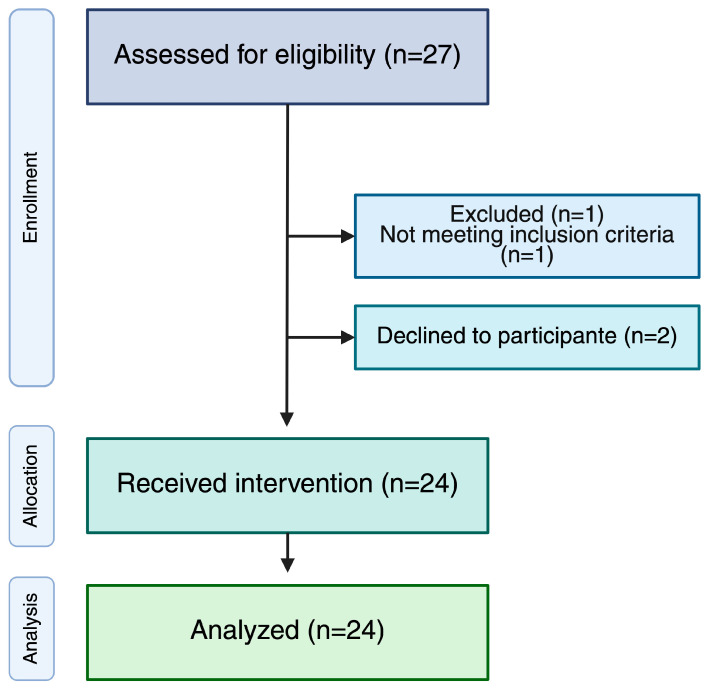
Flowchart diagram.

Of 27 eligible children and adolescents, 24 participants were enrolled in the study (see Figure 1). Participants (12 females) were 14 ± 5 years old, had a height of 140.9 ± 19.4 cm, and weight 36.5 ± 13.8 kg (see Table 1). Fourteen children had cerebral palsy, and all the participants had moderate-to-severe cognitive impairments. According to the GMFCS, nine participants were classified at level IV and 15 at level V. No adverse events occurred during the intervention.

### 3.2. Muscle Activity

The effects of passive bike on muscle activity of the 24 participants are shown in Table 2 and Table 3. Passive wheelchair bike ride resulted in an increase in muscle activity in the RLT, LUT, and LLT, with a delta increase ranging from 97% to 112%. The RST showed a significant increase from the resting phase (32.67 ± 31.54 µV) to the passive ride (42.57 ± 28.31 µV) (*p* = 0.04). The LUT also had a significant increase from resting (22.38 ± 17.00 µV) to passive ride (40.58 ± 42.40 µV) (*p* = 0.008). The LLT activity increased, but not significantly (*p* = 0.063). Significant changes in maximum activity were observed for the RST (*p* = 0.05) and the LLT (*p* = 0.038). The LUT showed a highly significant increase (*p* = 0.001) from rest (161.70 ± 130.13 µV) to bike ride (287.35 ± 187.93 µV). The RLT showed no differences in mean and maximum activity between the time points.

### 3.3. Cardiorespiratory Response

There were no differences in VO_2_ (*p* = *0.253*) during bike ride [4.30 ± 1.18 mL/(kg·min)] compared to rest condition [5.56 ± 2.21 mL/(kg·min)], for the 11 participants who tolerated the VO2 Master. This also occurred with the other measurements related to cardiorespiratory response (i.e., respiratory frequency, tidal volume, minute ventilation and equivalent of oxygen consumption). The results for all the outcomes are shown in Table 4.

### 3.4. Autonomic Nervous System Response

The HRV results of 18 participants are presented in Table 5 and Table 6. While an increase in STD HR was observed between resting time (5.92 ± 3.53 ms) and bike ride (11.2 ± 21.35 ms), this change did not reach statistical significance (*p* = 0.044). Likewise, no significant differences were detected in other frequency-domain variables. Analysis of time and group effects (GMFCS IV and V) revealed no statistically significant differences across the evaluated conditions.

## 4. Discussion

The results suggest that passive ride may lead to increased postural muscle activity. However, no significant changes were observed in VO_2_ in children and adolescents at GMFCS levels IV and V. Similarly, HRV analysis showed no substantial alterations in autonomic nervous system function, apart from a modest increase in STD HR.

Muscle activity provides valuable insights into the neuromuscular function and activation patterns in children and adolescents with GMFCS levels IV and V [30]. Previous studies have demonstrated that children with severe physical impairments display altered muscle activation and postural control [31]. Moreover, adequate muscle activation is essential for coordinating postural muscles during standing, directly impacting overall activity and participation [32]. In this study, trapezius muscle activation appeared to be increased in most regions, except for the RUT. This lower activation in the RUT could possibly be explained by structural deformities of the spine and impaired cephalic control, as it is possible that many participants may have leaned towards the right side of their wheelchair for support. This lack of activation of the RUT could represent a compensatory strategy aimed at mitigating the reduced ability to fine-tune postural muscle control. To our knowledge, no previous intervention has specifically measured muscle activity during passive ride in children and adolescents with GMFCS levels IV and V. However, enhancing muscle activation—particularly in postural muscles—holds the potential to improve motor function, stability, and overall participation in daily activities [33]. Therefore, passive ride may be an effective strategy to stimulate postural muscles, potentially leading to adaptations and improvements in muscle activity and structure in this population. Further research is required to confirm these potential benefits and to determine the long-term effects of such interventions.

The bike ride had no effect on the VO_2_ of the participants, reflecting the low metabolic demand of the intervention. This may be explained by the nature of the intervention, which, being passive bike ride, does not engage large muscle groups to generate sufficient metabolic demand. Passive ride did not appear to engage the dynamic muscles of the upper body, which have higher oxygen consumption than static muscles [34]. This could explain the lack of significant changes in VO_2_. Research indicates that non-ambulant children with cerebral palsy often have significantly lower VO_2_ max values compared to their ambulant peers, reflecting reduced aerobic capacity and overall fitness [35]. Furthermore, individuals with cerebral palsy classified as GMFCS levels IV–V often present limited cardiorespiratory responses due to chronic physical inactivity and impaired motor control, which may blunt aerobic adaptations [9]. Improving VO_2_ can enhance endurance, reduce fatigue, and promote better cardiovascular health [36]. Our results suggest that passive rides do not provide sufficient stimulus to acutely increase VO_2_. Identifying activities that can acutely increase it and provoke physiological adaptations are necessary, as passive ride alone appears insufficient for this purpose.

HRV is a marker of autonomic nervous system function and cardiovascular health. Our results showed no changes in any variables in time-frequency or time-domain parameters between resting time and passive wheelchair bike ride. However, authors suggest that children with CP have a lower cardiac autonomic system adaptation to exercise and activity [37] and this impairment may be related to prolonged sedentary behaviours [38]. Also, children with lower motor performance had lower HRV values [39]. The lack of HR and HRV response in our study may indicate that the stimuli was not enough to act on the autonomic system. In contrast, previous studies [40,41] suggest that certain forms of physical exercise and training improve cardiac autonomic regulation in this population by reducing HR and breathing rate, while increasing values of selected heart rate variability HRV parameters, such as the peak of LF during exercise.

While HRV is primarily HR-dependent, not all HRV indices follow this dependency. Pediatric patients with CP exhibited significantly higher resting HR and reduced HRV along with different responses (or no response) to selected movement maneuvers compared to typically developed children [38]. Individuals who required assistance for mobility showed decreased values of selected HRV parameters compared to their more independent peers, suggesting a different parasympathetic response of the autonomic nervous system [38,42,43]. Physical exercise positively influences cardiac autonomic regulation by reducing HR and respiratory rate and increasing values of selected HRV parameters [38]. Even though we observed an improvement in postural muscle activity during the intervention, the HRV parameters did not express this change. However, energy expenditure due to physical activity is not associated with any health-related index, as HRV, when physical activity is low [44]. In addition, previous studies suggest that HR autonomic regulation can be positively influenced by training in this population [40]. For this reason, further research is needed, as the degree of motor impairment in children correlates with their capacity to perform autonomic adjustments through the neuro-cardiac system [39].

This study has some limitations that need to be considered. Of the 24 participants, only 11 were able to use the gas analyzer, and in two participants, the Polar device did not fit due to their thoracic anatomy. In addition, spinal deformities in some participants affected the placement of the sEMG electrodes which was adjusted, preventing compliance with the SENIAM recommendations. Finnaly, this model of study cannot blind assesors of variables nor assesors of the intervention, and limits the found evidence. A key strength of this study is its comprehensive focus on a highly underrepresented population–children and adolescents with disabilities at GMFCS levels IV and V—providing valuable insights that can improve clinical care and quality of life for this population. By employing methods such as measuring muscle activity during passive ride using sEMG, assessing autonomic function through HRV, and including VO_2_ measurements, the study offers objective and quantifiable data that enhance the reliability of the findings. The inclusion of VO_2_ adds depth to understanding metabolic responses and aerobic capacity in this group.

## 5. Conclusions

This study suggests that a passive wheelchair bike ride increases trapezius muscle activity compared to conditions in children and adolescents with disabilities at GMFCS levels IV and V. These findings offer preliminary evidence that passive ride bike could be a useful intervention for improving fitness and neuromuscular function in this population. However, further studies are needed to confirm its effects on body structure and function, including parameters such as HR and VO_2_, and to investigate the potential long-term benefits of passive wheelchair ride bike interventions.

## Figures and Tables

**Table 1:** Descriptives of the participants.

Variables (mean ± SD)	Total	GMFCS IV	GMFCS V
**Sex** (n, % females)	24, 50%	9, 66%	15, 40%
**Age** (years)	14.42 ± 5.09	14.56 ± 5.43	14.33 ± 5.07
**Height** (cm)	140.92 ± 19.41	139.89 ± 18.84	141.53 ± 20.37
**Weight** (kg)	36.45 ± 13.80	34.34 ± 14.66	37.71 ± 13.62
**Cognitive impairment** Moderate (n, **%**) Severe (n, **%**)	7, 29% 17, 71%	3, 33% 6, 66%	4, 27% 11, 73%

Abbreviations: GMFCS: Gross Motor Function Classification Measure; SD: Standard Deviation.

**Table 2:** Effects of passive wheelchair bike ride on mean muscle activity in non-ambulant children with disabilities (GMFCS Levels IV–V).

Trapezius Activity Mean (n = 24)	Baseline (Resting Condition)	During Activity (Passive Wheelchair Bike Ride)	*p*-Value	Group-by-Time	Increase from Resting (Δ%)
**Right lower (µW)** (mean ± SD) [median (1st–3rd quartile)]	32.67 ± 31.54 17.77 (11.60–47.45)	42.57 ± 28.31 35.47 (21.79–55.06)	***0.040***	*0.682*	112.49
**Left lower (µW)** (mean ± SD) [median (1st–3rd quartile)]	26.12 ± 23.38 18.55 (10.52–27.63)	38.13 ± 32.07 30.06 (17.06–44.10)	*0.063*	*0.201*	100.66
**Left upper (µW)** (mean ± SD) [median (1st–3rd quartile)]	22.38 ± 17.00 17.85 (10.90–26.72)	40.58 ± 42.40 31.08 (18.08–42.19)	***0.008***	*0.277*	141.89
**Right upper (µW) (n with data = 23)** (mean ± SD) [median (1st–3rd quartile)]	22.33 ± 22.18 15.56 (7.60–27.56)	42.12 ± 59.18 26.36 (12.24–29.08)	*0.190*	*0.511*	96.68

Abbreviations: SD, Standard Deviation. Group-by-time analysis was made between Level IV and Level V on GMFCS.

**Table 3:** Effects of passive wheelchair bike ride on maximum muscle activity in non-ambulant children with disabilities (GMFCS Levels IV–V).

Trapezius Activity Peak (n = 24)	Baseline (Resting Condition)	During Activity (Passive Wheelchair Bike Ride)	Group-by-Time	*p*-Value
**Right lower (µW)** (mean ± SD) [median (1st–3rd quartile)]	186.93 ± 202.50 128.82 (66.26–238.89)	338.26 ± 198.99 327.98 (203.77–440.57)	*0.512*	***0.050***
**Left lower (µW)** (mean ± SD) [median (1st–3rd quartile)]	164.40 ± 126.15 116.82 (64.77–239.86)	267.14 ± 186.60 243.09 (126.39–419.04)	*0.918*	***0.038***
**Left upper (µW)** (mean ± SD) [median (1st–3rd quartile)]	161.70 ± 130.13 110.52 (69.95–244.69)	287.35 ± 187.93 267.64 (188.09–315.02)	*0.739*	***0.001***
**Right upper (µW) (n with data base = 23)** (mean ± SD) [median (1st–3rd quartile)]	234.07 ± 258.72 96.81 (62.80–304.75)	297.67 ± 196.99 271.81 (151.78–349.20)	*0.648*	*0.107*

Abbreviations: SD, Standard Deviation. Group-by-time analysis was made between Level IV and Level V on GMFCS.

**Table 4:** Effects of Passive Wheelchair Ride Bike on Cardiorespiratory Response in non-ambulant children with disabilities (GMFCS Levels IV–V).

Cardiorespiratory Response (n = 9)	Baseline (Resting Condition)	During Activity (Passive Wheelchair Bike Ride)	*p*-Value	Increase from Resting (Δ%)
**VO2 (mL/kg.min** **(n with data = 6)** (mean ± SD) [median (1st–3rd quartile)]	4.30 ± 1.18 4.06 (3.34–4.49)	5.56 ± 2.21 4.92 (3.49–5.74)	*0.253*	33.94
**HR (bpm) (n with data = 9)** (mean ± SD) [median (1st–3rd quartile)]	101.62 ± 13.42 101.62 (88.37–110.41)	111.57 ± 25.24 107.59 (97.12–116,56)	*0.126*	9.05
**Rf (breaths/min) (n with data = 9)** (mean ± SD) [median (1st–3rd quartile)]	21.10 ± 2.70 20.07 (19.24–21.10)	25.50 ± 5.53 24.99 (22.39–27.12)	*0.124*	24.39
**Tv[L] (n with data = 9)** (mean ± SD) [median (1st–3rd quartile)]	0.41 ± 0.07 0.41 (0.33–0.43)	0.46 ± 0.14 0.44 (0.34–0.48)	*0.274*	15.73
**Ve[L/min] (n with data = 9)** (mean ± SD) [median (1st–3rd quartile)]	8.53 ± 1.89 8.38 (6.95–8.59)	12.03 ± 6.01 10.14 (8.58–12.03)	*0.160*	47.96
**EqO2 (mL/kg·min (n with data = 6)** (mean ± SD) [median (1st–3rd quartile)]	39.65 ± 13.48 37.09 (24.72–42.15)	56.00 ± 37.49 39.55 (37.49–44.25)	*0.345*	23.92
**RR[ms] (n with data = 8)** (mean ± SD) [median (1st–3rd quartile)]	611.37 ± 96.77 587.47 (521.92–628.36)	581.46 ± 141.84 545.28 (479.30–603.26)	*0.330*	−5.71

Abbreviations: EqO2, Equivalent of Oxygen consumption; Rf, Respiratory frequency; RR, Intervals between R-wave peaks; VO2, Volume of Oxygen consumption; SD, Standard Deviation; Tv, Tidal Volume; Ve, Minute ventilation.

**Table 5:** Effects of Passive Wheelchair Ride Bike on Time-Domain Heart Rate Variability in non-ambulant children with disabilities (GMFCS Levels IV–V).

Autonomic Nervous System Response (n = 18)	Baseline (Resting Condition)	During Activity (Passive Wheelchair Bike Ride)	*p*-Value
**RR (ms)** (mean ± SD) [median (1st–3rd quartile)]	568.41 ± 76.33 568.58 (516.29–595.29)	651.72 ± 257.48 553.76 (510.66–745.43)	*0.286*
**SDNN (ms)** (mean ± SD) [median (1st–3rd quartile)]	31.08 ± 14.59 27.01 (19.00–44.89)	48.19 ± 42.90 33.57 (24.92–52.23)	*0.157*
**HR (beats/min)** (mean ± SD) [median (1st–3rd quartile)]	107.95 ± 16.85 105.53 (100.79–116.21)	100.64 ± 23.55 108.36 (80.65–117.50)	*0.252*
**STDHR (beats/min)** (mean ± SD) [median (1st–3rd quartile)]	5.92 ± 3.53 4.86 (3.86–7.60)	11.24 ± 21.35 5.46 (4.95–7.66)	*0.044*
**Minimum HR (beats/min)** (mean ± SD) [median (1st–3rd quartile)]	85.62 ± 24.55 88.92 (81.77–100.02)	79.84 ± 24.85 86.57 (65.83–97.65)	*0.616*
**Maximum HR (beats/min)** (mean ± SD) [median (1st–3rd quartile)]	133.56 ± 29.60 129.21 (112.52–138.19)	144.99 ± 56.94 136.20 (107.84–151.64)	*0.267*
**RMSSD (ms)** (mean ± SD) [median (1st–3rd quartile)]	25.41 ± 13.37 24.44 (13.21–32.43)	37.97 ± 25.68 31.15 (17.51–56.73)	*0.018*

Abbreviations: HR, heart rate; min, minutes; RR, intervals between R-wave peaks; SD, standard deviation; SDNN, standard deviation of NN (Normal-to-Normal) intervals; STDHR, Standard Deviation of the heart rate. Significant *p*-value was adjusted (*p* < 0.003) with Bonferroni effect of 16 variables.

**Table 6:** Effects of Passive Wheelchair Ride Bike on Frequency-Domain Heart Rate Variability in non-ambulant children with disabilities (GMFCS Levels IV–V).

Autonomic Nervous System Response (n = 18)	Baseline (Resting Condition)	During activity (Passive Wheelchair Bike Ride)	*p*-Value
VLF **Peak (Hz)** (mean ± SD) [median (1st–3rd quartile)]	0.04 ± 0.00 0.04 (0.03–0.04)	0.03 ± 0.00 0.04 (0.03–0.04)	*0.620*
**Power (ms^2^)** (mean ± SD) [median (1st–3rd quartile)]	86.35 ± 75.32 65.32 (17.84–94.30)	1123.68 ± 3068.60 71.28 (32.41–212.63)	*0.446*
**Power (%)** (mean ± SD) [median (1st–3rd quartile)]	11.54 ± 8.78 8.77 (4.57–15.35)	13.49 ± 13.59 8.71 (7.56–14.48)	*0.948*
LF **Peak (Hz)** (mean ± SD) [median (1st–3rd quartile)]	0.07 ± 0.03 0.06 (0.05–0.08)	0.08 ± 0.03 0.08 (0.05–0.11)	*0.193*
**Power (ms^2^)** (mean ± SD) [median (1st–3rd quartile)]	648.78 ± 699.86 353.01 (175.09–864.83)	1650.84 ± 2994.47 509.04 (266.69–828.71)	*0.446*
**Power (%)** (mean ± SD) [median (1st–3rd quartile)]	56.87 ± 15.20 53.69 (45.78–66.60)	55.84 ± 15.49 57.61 (47.58–72.53)	*0.806*
HF **Peak (Hz)** (mean ± SD) [median (1st–3rd quartile)]	0.23 ± 0.07 0.21 (0.16–0.28)	0.22 ± 0.08 0.18 (0.16–0.29)	*1.000*
**Power (ms^2^)** (mean ± SD) [median (1st–3rd quartile)]	411.64 ± 377.85 273.00 (48.41–533.27)	723.65 ± 906.27 299.53 (149.70–840.95)	*0.231*
**Power (%)** (mean ± SD) [median (1st–3rd quartile)]	31.55 ± 16.31 30.42 (16.43–45.52)	30.62 ± 18.01 29.41 (12.84–42.64)	*0.871*

Abbreviations: HF, High Frequency; LF, low frequency; VLF, very low frequency. Significant *p*-value was adjusted (*p* < 0.003) with Bonferroni effect of 16 variables.

## Data Availability

Data is contained within the article.

## Abbreviations

The following abbreviations are used in this manuscript:

GMFCS	Gross Motor Function Classification System
WHO	World Health Organization
VO_2_	Oxygen consumption
HRV	Heart rate variability
sEMG	Surface electromyography
ICF-CY	International classification of functioning, disability, and health for children and youth
ICF	International Classification of Functioning, Disability, and health
LUT	Left upper trapezius
RUT	Right upper trapezius
LLT	Left lower trapezius
RLT	Right lower trapezius
SENIAM	Surface Electromyography for the non-invasive assessment of muscles
HR	Heart rate
SDNN	Standard deviation of normal-to-normal intervals
RMSSD	Root mean square of the successive differences
STDHR	Standard deviation of the heart rate
SPSS	Statistical Package for Social Sciences
